# Development of an HIV vaccine using a vesicular stomatitis virus vector expressing designer HIV-1 envelope glycoproteins to enhance humoral responses

**DOI:** 10.1186/s12981-017-0179-2

**Published:** 2017-09-12

**Authors:** Trina Racine, Gary P. Kobinger, Eric J. Arts

**Affiliations:** 1 Department of Medical Microbiology, University of Manitoba, Winnipeg, Canada; 2 Centre de Recherche en Infectiologie, Centre Hospitalier Universitaire de Québec, Université Laval, Québec City, QC Canada; 3 Department of Immunology, University of Manitoba, Winnipeg, Canada; 4 Department of Pathology and Laboratory Medicine, University of Pennsylvania School of Medicine, Philadelphia, PA USA; 5 Department of Microbiology and Immunology, Western University, London, Canada

**Keywords:** HIV, Vesicular stomatitis virus, Vaccine, Env antigens

## Abstract

Vesicular stomatitis virus (VSV), like many other Rhabdoviruses, have become the focus of intense research over the past couple of decades based on their suitability as vaccine vectors, transient gene delivery systems, and as oncolytic viruses for cancer therapy. VSV as a vaccine vector platform has multiple advantages over more traditional viral vectors including low level, non-pathogenic replication in diverse cell types, ability to induce both humoral and cell-mediate immune responses, and the remarkable expression of foreign proteins cloned into multiple intergenic sites in the VSV genome. The utility and safety of VSV as a vaccine vector was recently demonstrated near the end of the recent Ebola outbreak in West Africa where VSV pseudotyped with the Ebola virus (EBOV) glycoprotein was proven safe in humans and provided protective efficacy against EBOV in a human phase III clinical trial. A team of Canadian scientists, led by Dr. Gary Kobinger, is now working with International AIDS Vaccine Initiative (IAVI) in developing a VSV-based HIV vaccine that will combine unique Canadian research on the HIV-1 Env glycoprotein and on the VSV vaccine vector. The goal of this collaboration is to develop a vaccine with a robust and potent anti-HIV immune response with an emphasis on generating quality antibodies to protect against HIV challenges.

## Background

Over 36 million people are living with HIV worldwide and despite the rollout of effective HIV treatments, there are still 1.8 million new infections a year that could be prevented by an effective vaccine [1]. Over the last three decades only four preventative HIV vaccine concepts have been tested for clinical efficacy. Monomeric HIV envelope glycoproteins (GPs) (AIDSVAX B/E gp120) failed to demonstrate protection against HIV infection in clinical trials and demonstrated poor antibody response to the trimeric envelope GP on native HIV [2, 3]. The STEP trial was aimed at stimulating strong CD8+ T cell responses to HIV infected cells by vaccinating with a recombinant adenovirus serotype 5 (rAd5) vector-based vaccine expressing the HIV-1 internal proteins gag/pol/nef. This human trial was stopped due to inefficacy and increased HIV-1 acquisition in vaccinated subgroups. The subsequent HVTN505 trial was halted because priming with DNA vaccines expressing gag/pol/nef/env and boosting with rAd5 vectors expressing gag/pol/env did not protect against HIV acquisition or lower HIV-1 RNA in breakthrough infections [4]. While this vaccine afforded partial protection against low stringency simian immunodeficiency viruse (SIV) challenges (SIVsmE660) in rhesus macaques, it failed to protect against high stringency SIV challenges (SIVmac251) [5], stressing the importance of performing preclinical studies of HIV-1 vaccines in stringent preclinical NHP challenge models.

So far, the only HIV vaccine efficacy trial to show promise was the RV144 trial conducted in Thailand where a prime with a canarypox vector (ALVAC) expressing gag/pol/nef and boost with a recombinant HIV gp120 [6] initially resulted in 60% efficacy in infection reduction within the first year of the study. However, this efficacy was subsequently reduced to a modest vaccine efficacy of 31%. Interestingly, higher titers of non-neutralizing IgG antibodies against the V1/V2 region of the envelope protein showed greater association with reduced infection in the RV144 trial than cytolytic CD8+ T cell responses whereas broadly neutralizing antibodies (bNAb) were rarely observed [7]. However, the modest success of RV144 does not discount the importance of bNAb in protection considering that administration of bNAbs to macaques have provided the best immune-associated protection from SHIV infection to date [8, 9], better than previous vaccine candidates. Experience from the STEP, RV144 and HVTN505 trials has set a path for novel vaccine approaches capable of generating more robust immunity against HIV.

**Fig. 1:**
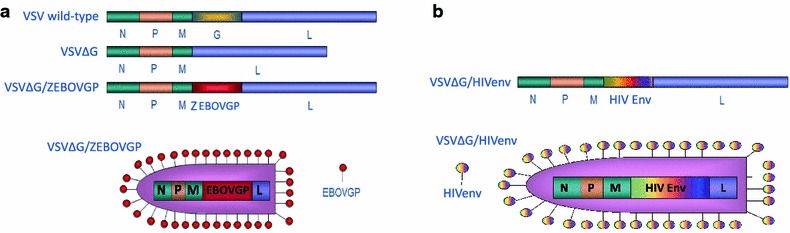
**a** Schematic drawing of the wild-type VSV genome (VSV wild-type), the VSV genome lacking the G protein (VSV∆G) and the recombinant form of the genome with the Ebola GP inserted in place of VSV G (VSV∆G/EBOVGP), along with an illustration depicting the rVSV∆G/EBOVGP vaccine vector. **b** Schematic drawing of the recombinant VSV genome with an HIV Env gene inserted in place of the VSV G protein, along with an illustration depicting the VSV∆G/HIVenv vaccine vector

Replication competent vaccines have traditionally generated a wider repertoire of immune defenses than their non-replicating counterparts, a condition that may provide enhanced protection to a highly diverse HIV-1 pathogen. The vesicular stomatitis virus (VSV) platform is a replication competent vaccine that has been shown to generate both cell-mediated and humoral immunity to expressed foreign antigens. Notably, this vector, pseudotyped with the Ebola virus (EBOV) GP (Fig. 1a) is safe to administer to humans [10] and, importantly, has shown protective efficacy against Ebola virus in a human phase III clinical trial [11]. Lack of pre-existing immunity to this vector helps overcome many of the drawbacks and safety concerns that arose with the Ad5-based vaccine [12]. Importantly, Dr. Chris Parks of the International AIDS Vaccine Initiative (IAVI) has recently presented preclinical vaccine studies testing a VSV vector pseudotyped with HIV-1 Env spikes (VSVΔG/HIVenv) that resulted in 67% protection in a non-human primate model of infection [13]. Despite this impressive protection, the vector was difficult to propagate in vitro and relied on CD4 and CCR5 receptors in cell lines for vector expansion [14]. When used for immunization the VSVΔG/HIVenv could induce anti-Env binding antibodies and cell-mediated immune responses in mice [15] and macaques [13], however there is no clear evidence yet for a neutralizing antibody response. We hope to improve the low surface expression of the HIV-1 envelope on the VSV vector, which remains a challenge for maximizing immunogenicity and for cost effective vaccine manufacturing. We are currently applying state-of-the-art cell culture technologies to develop improved cell lines, analytical methods, integrated production and purification technologies and formulation of HIV vectored vaccines. Our research approach involves enhancing the immunogenicity of the VSVΔG/HIVenv vaccine through the use of “designer” Env glycoproteins (Fig. 1b), by stabilizing Env surface expression on VSV with Ebola GP. We will also eliminate the vaccine vector’s dependency on CD4+/CCR5+ cells for replication through the introduction of Ebola GP in *cis* and by continuing our development of new processes for the generation of high vaccine titers compatible with preclinical primate model studies and further clinical progression in human clinical trials.

## Experience with VSV-EBOV GP vaccine

Vesicular stomatitis virus has been used as a vaccine vector for more than two decades for a range of infectious diseases including influenza virus [16] and Hepatitis C virus [17]. The first report of VSV pseudotyped with the Ebola GP was not for use as a vaccine, but instead as a system for the functional analysis of the Ebola GP since the highly pathogenic nature of this virus would normally require a containment level 4 (CL-4) laboratory for such analyses [18]. Subsequent work performed by Heinz Feldmann and colleagues at the National Microbiology Laboratory in Winnipeg, Canada, resulted in the development of a replication-competent system to study the function of the transmembrane proteins of various CL-4 pathogens [19]. This study by Garbutt and colleagues [19] was the first attempt to utilize the recombinant VSV vector to induce protection from lethal EBOV challenge in a mouse model. The utility of VSV as a vaccine vector for EBOV infection was then realized the following year with the publication of the Jones et al. [20] paper showing 100% protection of non-human primates following immunization with a single dose of the attenuated replication-competent rVSV∆G/ZEBOVGP vaccine. Subsequent to the publication of these results and thanks to a Government of Canada grant to the Public Health Agency of Canada, the rVSV∆G/ZEBOVGP vaccine was manufactured under current Good Manufacturing Practices and was available during the 2013–2016 West Africa Ebola outbreak for clinical testing where the safety [10, 21] and efficacy [11] of the rVSV∆G/ZEBOVGP vaccine was demonstrated. Under the guidance of Merck, this vaccine is now undergoing licensure and will hopefully be immediately available to help curb any future outbreak.

In addition to its demonstrated efficacy, the rVSV∆G/ZEBOVGP vaccine also induces long-term protection in mice and guinea pigs [22], a feature that would be very useful in a HIV vaccine. Also of importance, the Kobinger lab has successfully demonstrated the versatility of the VSV vector as a multivalent vaccine candidate able to confer protection against multiple unrelated and highly virulent pathogens (Ebola virus and pandemic H5N1 influenza virus), without significantly compromising the efficacy of each individual component in a mouse model of infection [23].

## Challenges in developing a VSV-based HIV vaccine

**Fig. 2:**
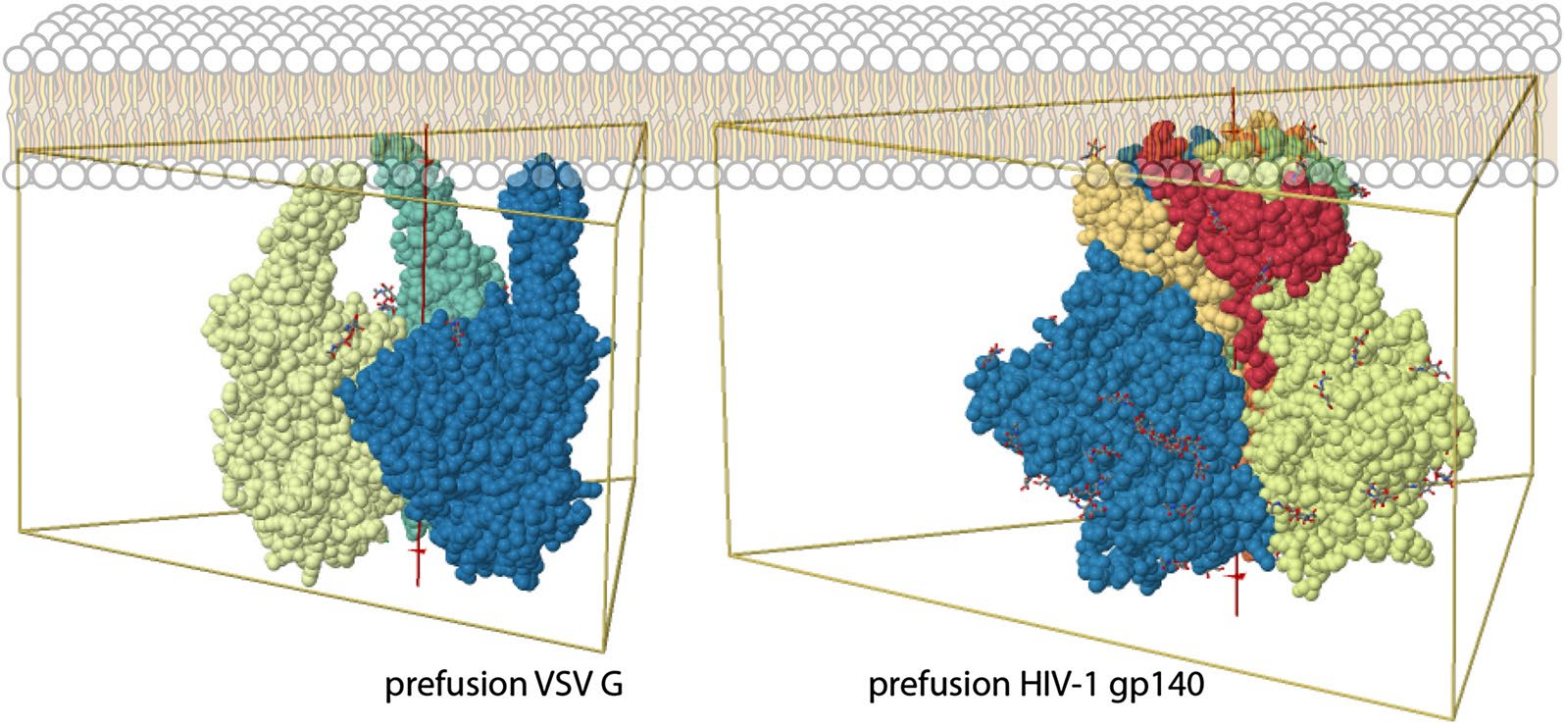
Schematic of the prefusion intermediate of VSV G glycoprotein structure (*left*) (PDB 5I2M) [36] and of the HIV Env gp140 structure (*right*) (PDB 4ZMJ) [37] in juxtaposition to a membrane

Unlike many other enveloped viruses, including VSV, HIV-1 is somewhat unique in the low density of virus-specific glycoprotein “spikes” on the surface of the virus particle exposed to the extracellular matrix. HIV-1 has approximately 10–20 trimer Env glycoprotein spikes per virion whereas even its closest relative, SIV, tends to have higher numbers of spikes, generally tenfold more. In contrast, VSV, a rhabdovirus of similar size to HIV-1 (70–130 nm) harbors at least 300 trimer glycoprotein (G) spikes or approximately 30-fold more spikes per viral envelop surface area than HIV. Despite the greater mass of the HIV-1 Env trimer (480 kDa) compared to the VSV G trimer (210 kDa), the prefusion state of HIV-1 Env trimer appears more compact and might suggest that on simple basis of stearic hindrance, less and not more VSV G trimer might be accommodated on the VSV particle as compared to Env trimer spikes on the HIV-1 particle (Fig. 2). This relative lack of functional trimers on the HIV surface is considered a serious impediment to the development of vaccines looking to simulate protective humoral immunity.

Of course, interaction of the viral matrix proteins (named M in VSV and MA in HIV-1), associations with cellular membrane proteins, composition of the lipid raft, transport of envelope glycoproteins to the cell membrane, and sites of viral budding can all play a role in incorporation of glycoprotein spikes into the virus particles. All of these factors likely differ between VSV and HIV such that pseudotyping with each other’s envelop spikes will likely result in variations in the levels of glycoprotein on the viral surface. As a consequence, it is not surprising that VSV particles are poorly pseudotyped by HIV-1 Env [14]. However, this same logic does not explain why VSV particles are efficiently pseudotyped by EBOV GP [13, 18]. Lorenz et al. [24] have shown that the membrane-proximal external region (MPER) of VSV G and HIV-1 Env can be interchanged without a loss in function. When this research group discovered poor packaging and expression of HIV-1 Env on the VSV vector surface, substitution of the HIV-1 gp41 MPER and transmembrane (TM) region with the analogous region in VSV G resulted in the appearance of a new HIV-1_gp120/VSV_G_TM chimera on the VSV vector particles. Considering these observations on the pseudotyping of VSV∆G particles with this HIV-1_gp120/VSV_G_TM chimera, we are now attempting to incorporate new HIV-1 Env chimeras into VSV particles. These chimeras will include the SIV MPER and TM domains, a similar membrane proximal and TM region of EBOV GP, and modification on the HIV-1 MPER/TM that might improve transport to the cell membrane and reduce Nef-mediated endocytosis. We hypothesize that these changes may improve the Env spike density on the vector surface and the presence of the SIV MPER and TM domains may induce the generation of more relevant/protective antibodies.

Aside from the difficulties in pseudotyping VSV with HIV-1 Env, our research field has generally struggled in identifying the best envelop glycoprotein as an immunogen, whether it be for a simple monomeric gp120, trimeric gp140, or for expression from various viral vectors. The general approach has been to use a native Env sequence of a laboratory or primary HIV-1 strain that adopts an unliganded conformation, which typically elicits non-neutralizing, anti-Env binding antibodies thus inducing limited protection from viral challenges in animal studies. Although no HIV vaccine to date has elicited bNAbs to HIV, many RNA virus infections in humans are cleared following or during acute infection by neutralizing antibodies. Likewise, preventative humoral-based vaccines inducing neutralizing antibodies are often associated with the best protection against these same viral infections. In support of this dogma, passive transfer of bNAb to macaques provided the best immune-related protection to SHIV infections to date [8, 9]. bNAbs have now been isolated from several HIV infected individuals at late stage infection and this topic has been reviewed by Ahmed et al. [25].  To elicit these all-mighty bNAbs, several research teams are using sequential vaccinations with mosaic Env vaccines or designer Env-based mimetics. SOSIP.BG505 Env gp140 trimers (derived from the subtype A BG505 Env with I559P) has been at the “heart” of screening and characterization of these bNAbs [6, 26], as well as designing/immunizing with a series of immunogens that may select for specific B cell clones with a propensity to produce bNAbs [27–29]. Nonetheless, with the rVSV∆G/ZEBOVGP vaccine [30] and the Thai HIV RV144 vaccine trials [6, 7], the levels of neutralizing antibodies did not fully correlate with protection suggesting that other antibody types or binding specificities may also provide antiviral activity related to antibody opsonization for phagocytosis, antibody-dependent cell-mediated cytotoxicity (ADCC), and antibody-mediated complement activity.

**Fig. 3:**
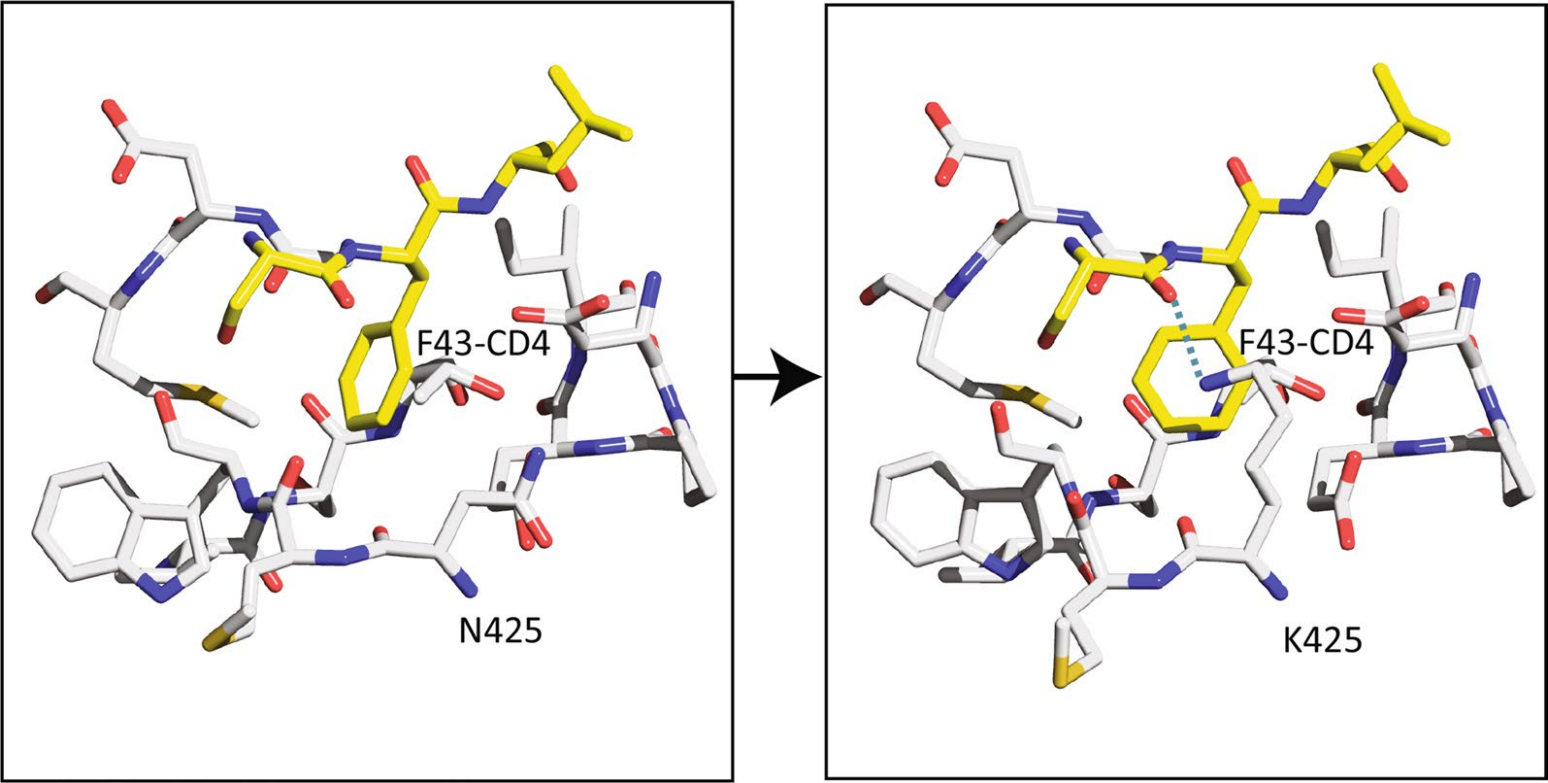
Schematic of gp120 HIV-1YU-2 complexed with CD4 and 412 Ab (PDB ID: 2QAD) [38] and modeled using the COOT program to contain the N425 to K mutation in gp120. The Nε of K425 can interact with residues from CD4 including a cation-π interaction with F43 of CD4 (This model was adapted from [35])

Our approach in Env selection is based on maximizing both binding antibodies for ADCC and for broad neutralization/inhibition of most HIV-1 strains. Most bNAbs target conserved epitopes in HIV Env that are typically hidden by the glycan “shield” [31, 32] and some are only exposed in an “open” conformation upon binding to CD4 [33, 34]. Our preliminary studies have described a naturally occurring Env gp120 polymorphism, K425, in a subtype A strain that demonstrates high binding affinity to CD4, greater host cell entry efficiency, higher replicative fitness, and resistance to Maraviroc [35], and finally, modelled to show enhanced CD4 binding due to formation of new H-bond between K425 and F43 of CD4 (Fig. 3). However, this naturally occurring variant is rarely found in HIV-infected individuals because K425 Env may also induce potent bNAbs (due to a natural SOSIP/open-like structure) leading to immediate self-elimination. Placing this K425 in the Env expressed by VSV may result in a stable immunogen that elicits bNAbs and in the case of new HIV-1 exposure, would provide protection and eliminate the possibility of escape as observed in concurrent HIV infections.

## Conclusions

We suspect that these designer Env antigens, expressed on the surface of a non-pathogenic but replicating vector such as VSV will elicit a potent anti-HIV Env antibody response and provide an effective or optimal protection against an HIV-1 challenge. Our research team, which includes Drs. Eric Arts, Blake Ball, Eric Cohen, Carole Creuzenet, Jimmy Dikeakos, Jerome Estaquier, Keith Fowke, Bruno Gaillet, Yong Gao, Alain Garnier, Renald Gilbert, Amine Kamen, Chil-Yong Kang, Gary Kobinger, Jamie Mann, Trina Racine, Michel Tremblay and Xiao-Jian Yao, in collaboration with IAVI and with the European AIDS Vaccine Initiative 2020 (EAVI2020) are preparing new VSV-HIV vaccines for animal testing starting in the fall of 2017 and with the hope of initiating human trials as early as 2019.

## Abbreviations

ADCC: antibody-dependent cell-mediated Cytotoxicity
bNAbs: broadly neutralizing antibodies
CL-4: containment level 4
EAVI2020: European AIDS Vaccine Initiative 2020
EBOV: Ebola Virus
GP: glycoprotein
IAVI: International AIDS Vaccine Initiative
MPER: membrane-proximal external region
rAd5: recombinant adenovirus serotype 5
SIV: simian immunodeficiency virus
TM: transmembrane
VSV: vesicular stomatitis virus
